# Performance and Safety of the Extravascular Implantable Cardioverter Defibrillator Through Long-Term Follow-Up: Final Results From the Pivotal Study

**DOI:** 10.1161/CIRCULATIONAHA.124.071795

**Published:** 2024-09-26

**Authors:** Paul Friedman, Francis Murgatroyd, Lucas V.A. Boersma, Jaimie Manlucu, Bradley P. Knight, Nicolas Clémenty, Christophe Leclercq, Anish Amin, Béla Merkely, Ulrika Maria Birgersdotter-Green, Joseph Yat Sun Chan, Mauro Biffi, Reinoud Elwin Knops, Gregory Engel, Ignacio Muñoz Carvajal, Laurence M. Epstein, Venkata Sagi, Jens Brock Johansen, Maciej Sterliński, Clemens Steinwender, Troy Hounshell, Richard Abben, Amy E. Thompson, Yan Zhang, Christopher Wiggenhorn, Sarah Willey, Ian Crozier

**Affiliations:** 1 Mayo Clinic, Rochester, MN (P.F.).; 2 King’s College Hospital, London, UK (F.M.).; 3 Cardiology Department of St. Antonius Hospital Nieuwegein, Netherlands (L.V.A.B.).; 4 London Health Sciences Centre, ON, Canada (J.M.).; 5 Northwestern University, Evanston, IL (B.P.K.).; 6 Clinique du Millenaire, Montpellier, France (N.C.).; 7 CHU de Rennes-Hôpital Pontchaillou, France (C.L.).; 8 Riverside Methodist Hospital, Columbus, OH (A.A.).; 9 Heart and Vascular Center, Semmelweis University, Budapest, Hungary (B.M.).; 10 University of California San Diego, San Diego (U.M.B.-G.).; 11 Prince of Wales Hospital, Chinese University of Hong Kong, China (J.Y.S.C.).; 12 Policlinico Sant’ Orsola–Malpighi, Bologna, Italy (M.B.).; 13 Amsterdam University Medical Centers Netherlands (L.V.A.B., R.E.K.).; 14 Palo Alto Medical Foundation, Redwood City, CA (G.E.).; 15 Hospital Universitario Reina Sofía, Córdoba, Spain (I.M.C.).; 16 Northwell Health, Manhasset, NY (L.M.E.).; 17 Baptist Health, Jacksonville, FL (V.S.).; 18 Odense Universitetshospital, Denmark (J.B.J.).; 19 I Klinika Zaburzeń Rytmu Serca/ Narodowy Instytut Kardiologii, Warszawa, Poland (M.S.).; 20 Kepler University Hospital, Linz, Austria (C.S.).; 21 Iowa Heart Center, West Des Moines (T.H.).; 22 Cardiovascular Institute of the South, Houma, LA (R.A.).; 23 Medtronic Inc, Mounds View, MN (A.E.T., Y.Z., C.W., S.W.).; 24 Christchurch Hospital, New Zealand (I.C.).

**Keywords:** arrhythmias, cardiac, cardiac pacing, artificial, death, sudden, cardiac, defibrillators, implantable, primary prevention, secondary prevention

## Abstract

**BACKGROUND:**

Substernal lead placement of the extravascular implantable cardioverter defibrillator (EV ICD) permits both defibrillation at thresholds similar to those seen with transvenous implantable cardioverter defibrillators and effective anti-tachycardia pacing (ATP) while avoiding the vasculature and associated complications. The global Pivotal study has shown the EV ICD system to be safe and effective through 6 months, but long-term experience has yet to be published. Our aim was to report the performance and safety of the EV ICD system throughout the study.

**METHODS:**

The EV ICD Pivotal study was a prospective, global, single-arm, premarket clinical study. Individuals with a Class I or IIa indication for a single-chamber implantable cardioverter defibrillator per guidelines were enrolled. Freedom from major system- or procedure-related complications and appropriate and inappropriate therapy rates were assessed through 3 years with the Kaplan-Meier method. ATP success was calculated from simple proportions.

**RESULTS:**

An implantation was attempted in 316 patients (25.3% female; 53.8±13.1 years of age; 81.6% primary prevention; left ventricular ejection fraction, 38.9±15.4%). Of 299 patients with a successful implantation, 24 experienced 82 spontaneous arrhythmic episodes that were appropriately treated with ATP only (38, 46.3%), shock only (34, 41.5%), or both (10, 12.2%) for a Kaplan-Meier–estimated rate of first any appropriate therapy of 9.2% at 3 years. ATP was successful in 77.1% (37/48) of episodes, and ATP use significantly increased from discharge to last follow-up visit (*P*<0.0001). Shock therapy was successful in 100% (27/27) of discrete, spontaneous ventricular arrhythmias. The inappropriate shock rates at 1 and 3 years were 9.8% and 17.5%, respectively, with P-wave oversensing the predominant cause. No major intraprocedural complications were reported, and the estimated freedom from system- or procedure-related major complications was 91.9% at 1 year and 89.0% at 3 years. The most common major complications were lead dislodgement (10 events; n=9 patients, 2.8%), postoperative wound or device pocket infection (n=8, 2.5%), and device inappropriate shock delivery (n=4, 1.3%). Twenty-four system revisions were performed as a result of major complications related to the EV ICD system or procedure.

**CONCLUSIONS:**

From implantation to study completion, the EV ICD Pivotal study demonstrated that a single integrated system with an extravascular lead placed in the substernal space maintains high ATP success, effective defibrillation, and a consistent safety profile.

**REGISTRATION:**

URL: https://www.clinicaltrials.gov; Unique identifier: NCT04060680.

**Clinical Perspective**

**What Is New?**

- The first extravascular defibrillation system with a substernal lead effectively delivers anti-tachycardia pacing, successfully terminating 77% of episodes in which anti-tachycardia pacing was used during a mean follow-up of 30.6 months.
- The extravascular implantable cardioverter defibrillator (EV ICD) system provides effective defibrillation therapy during long-term follow-up, terminating 100% of discrete, spontaneous ventricular arrhythmias.
- In this study, the EV ICD system had a rate of freedom from system- or procedure-related major complications of 89% at 3 years. No major intraprocedural complications occurred, nor were there any unique major implantable cardioverter defibrillator complications related to the EV ICD system or procedure from implantation through final follow-up.

**What Are the Clinical Implications?**

- The EV ICD system provides effective treatment of potentially life-threatening ventricular arrhythmias in the long term, avoiding shocks in nearly half of spontaneous ventricular tachycardia or ventricular fibrillation episodes because of the availability of anti-tachycardia pacing.
- Only a minority of complications occurred after the previously reported 6-month follow-up, demonstrating the safety of the EV ICD over longer time frames in the Pivotal study.

Implantable cardioverter defibrillators (ICDs) are established therapy for reducing the incidence of sudden cardiac death in at-risk populations.^1,2^ Traditional transvenous placement of an ICD lead can result in serious complications in both the short and long term, including vascular injury, venous obstructions, systemic infections, cardiac perforation, and complications during chronic lead extraction.^3–5^ The subcutaneous ICD (S-ICD) was introduced as an alternative to transvenous systems, placing the lead between the skin and the sternum.^6,7^ By avoiding the vasculature, the S-ICD reduces the number and severity of complications compared with transvenous ICDs while effectively terminating ventricular arrhythmias.^8^ However, it does not provide anti-tachycardia pacing (ATP), and its location outside the chest wall necessitates a larger generator for higher-energy shocks, which can affect battery longevity.^6,9^

The extravascular ICD (EV ICD) was developed to provide the benefits of circumventing the vasculature while retaining many of the capabilities of a transvenous ICD system. Substernal lead placement of the EV ICD allows ATP and defibrillation therapy from a single device, with a size, projected longevity, and defibrillation threshold similar to those of transvenous systems while being outside the vasculature.^10–12^ Results from the first in-human pilot study, conducted in a small cohort of patients, showed that the EV ICD system could be safely implanted and could deliver effective defibrillation.^10,13^ These findings were validated in the subsequent Pivotal study in which the primary results exceeded safety and efficacy criteria in a large, global population through 6 months of follow-up.^11^ We now report the safety and efficacy of the EV ICD system through the extended follow-up of the Pivotal study.

## METHODS

The data underlying these results will not be made available to other researchers for the purposes of reproducing the results or replicating the procedures.

### Study Design and Patient Selection

The EV ICD Pivotal study design has been described in detail previously.^14^ Briefly, it was a prospective, global, multicenter, single-arm, nonrandomized, premarket approval study that enrolled patients between 2019 and 2021 with a class I or IIa indication for an ICD as recommended by international guidelines.^15^ Patients were excluded if they had a bradycardia or cardiac resynchronization therapy pacing indication or had undergone prior sternotomy (full exclusion criteria listed in Table S1). Patients with a successful implantation attempt were followed up and assessed at prehospital discharge (PHD), 2 weeks, 3 months, 6 months, and every 6 months thereafter until exited from the study. The study protocol was approved by ethics committees at each participating site, and written informed consent was provided by all study patients.

### Objectives

The primary safety end point of the Pivotal study was freedom from system- or procedure-related major complications at 6 months, and that same end point was assessed in this analysis through 3 years. We also examined the appropriate and inappropriate therapy rates through 3 years. Data that were collected through case report forms at each follow-up visit, including reporting of ATP programming and electrical performance (R-wave amplitude, pacing capture threshold, pulse width, and impedance), are summarized through 3 years or the last available follow-up. The total number of treated episodes (appropriate and inappropriate), ATP success, shock success, total major and minor complications, and system revisions were generated with data through the last available follow-up. Shock success was calculated for discrete, spontaneous episodes; success for shocks delivered as part of a ventricular tachycardia (VT) storm (≥3 episodes within 24 hours) is summarized separately.

### Adverse Event and Episode Adjudication

All adverse events were adjudicated by a Clinical Events Committee. The committee determined if the event was related to the EV ICD system or procedure and, for those that were related, if it was a major complication, a minor complication, or an observation. All induced episodes, all device-detected spontaneous episodes (excluding nonsustained VTs of ≤5 seconds in duration), and all episodes receiving device therapy were adjudicated as appropriate or inappropriate by an Episode Review Committee. Several events required readjudication during the study; additional details can be found in the Supplemental Methods.

### Statistical Analysis

Descriptive statistics are reported as mean±SD or median for continuous variables and frequency and percentage for categorical variables. The freedom from major complication rate, appropriate therapy rate, and inappropriate therapy rate were generated with the Kaplan-Meier method. ATP success rate was calculated with both simple proportions and the generalized estimating equations method to account for within-patient correlation (Supplemental Results). Change in ATP programming comparing PHD with last follow-up was assessed with the McNemar test. All statistical analyses were performed with SAS version 9.4 (SAS Institute, Cary, NC).

## RESULTS

### Baseline Characteristics

A total of 356 patients were enrolled, of whom 316 underwent an implantation attempt (25.3% female, 53.8±13.1 years of age). Forty patients exited before undergoing an implantation attempt, largely for administrative reasons (Figure S1). Patients with an implantation attempt had a mean left ventricular ejection fraction of 38.9±15.4%, and a majority (81.6%) underwent implantation for a primary prevention indication. A full list of patient baseline characteristics is provided in Table 1.

**Table 1.**
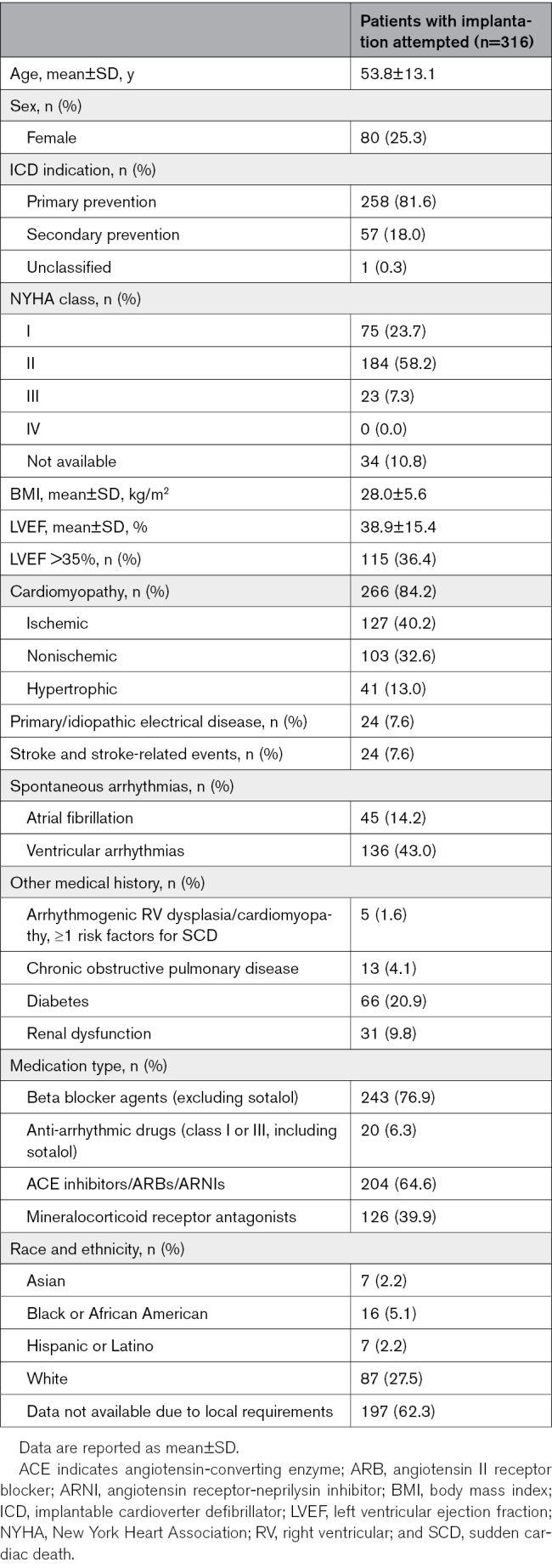
Baseline Patient Characteristics

### Follow-Up

Implantation attempt was successful in 299 patients, and those patients were followed up for an average of 30.6±8.5 months. The first implantation occurred in September 2019, and the last patient exited the study in January 2024. All subjects were followed up for a minimum of 2 years, unless they exited before study closure for other reasons; the longest follow-up was 4.2 years after implantation, and one patient was lost to follow-up (Figure S1).

### Appropriate Therapy

**Figure 1.**
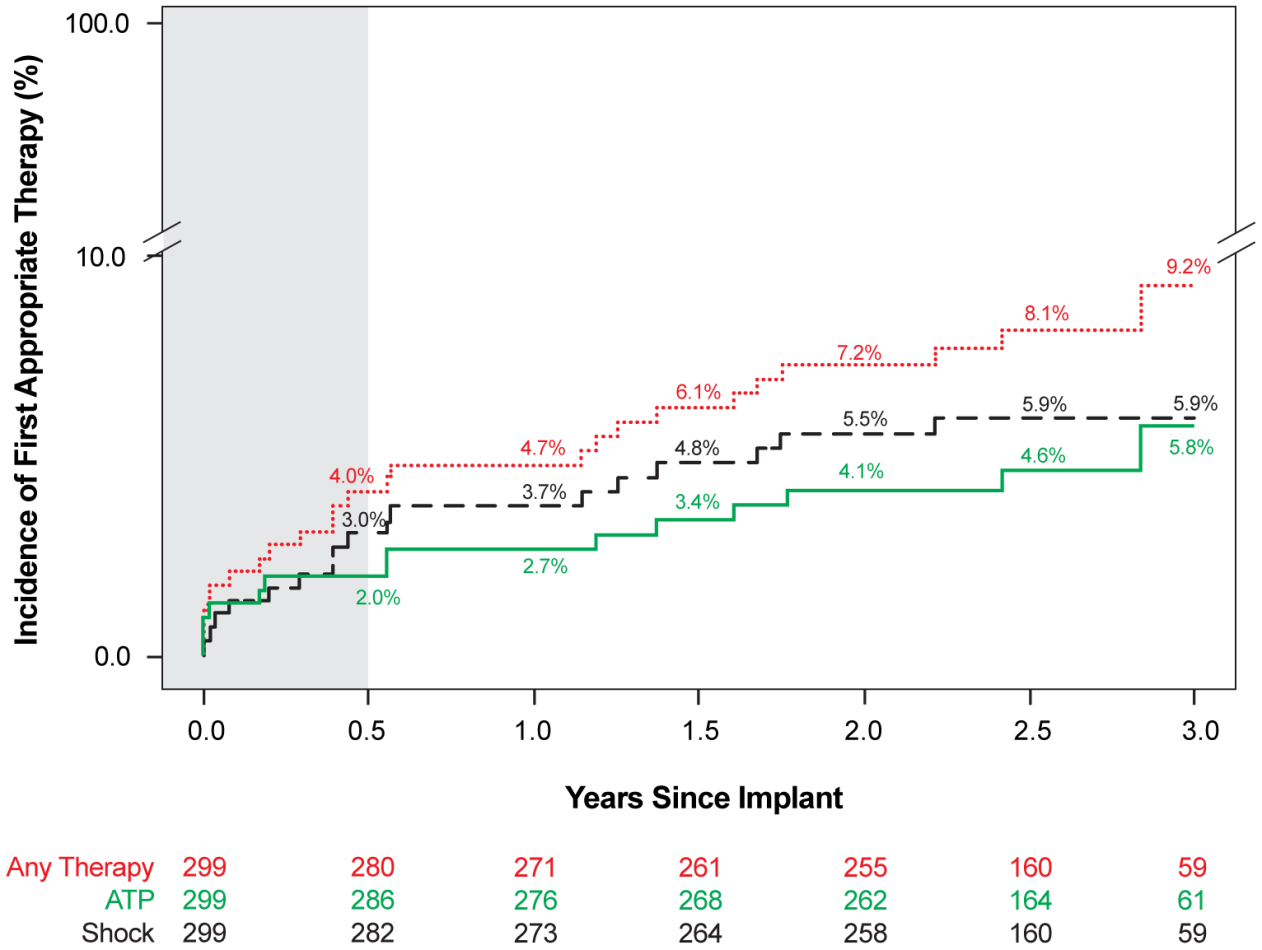
**Cumulative rate of first appropriate therapy through 3 years.** Kaplan-Meier–estimated time to first appropriate shock, anti-tachycardia pacing (ATP), or any therapy through 3 years. Number at risk is represented below in red (any therapy), green (ATP), and black (shock). The 6-month time frame is shaded in gray.

Through all of the follow-up, appropriate therapy was received by 24 patients for 82 spontaneous arrhythmic episodes, with 38 episodes (46.3%) receiving ATP only, 34 (41.5%) receiving shock only, and 10 (12.2%) receiving both ATP and shock. Among 27 discrete, spontaneous episodes (n=17 patients) treated with shock, all 27 (100%) were successfully converted to normal sinus rhythm. In addition, 4 patients received shock therapy for 17 episodes during VT storm, with 16 being successful. In one episode, the outcome could not be determined because of device storage limitations, but the patient was hospitalized, at which point the arrhythmia was resolved. The Kaplan-Meier–estimated rate of first appropriate therapy (ATP, shock, or both) was 9.2% at 3 years (Figure 1).

### Characterization of ATP

There were 48 monomorphic VT episodes (n=14 patients) that received appropriate ATP therapy, with 37 episodes (n=9 patients) being successfully terminated by ATP for a success rate of 77.1%. ATP was nominally “off” in the device, and the proportion of patients who were reported to have ATP programmed “on” significantly increased from 66.8% at PHD to 81.2% at the last study visit (*P*<0.0001; Table S2). At 2 years, ATP was programmed “off” in 2.8% of patients because of pacing sensation during in-clinic electrical testing; however, no patient that received successful ambulatory ATP subsequently had it programmed “off.”

### Safety

**Figure 2.**
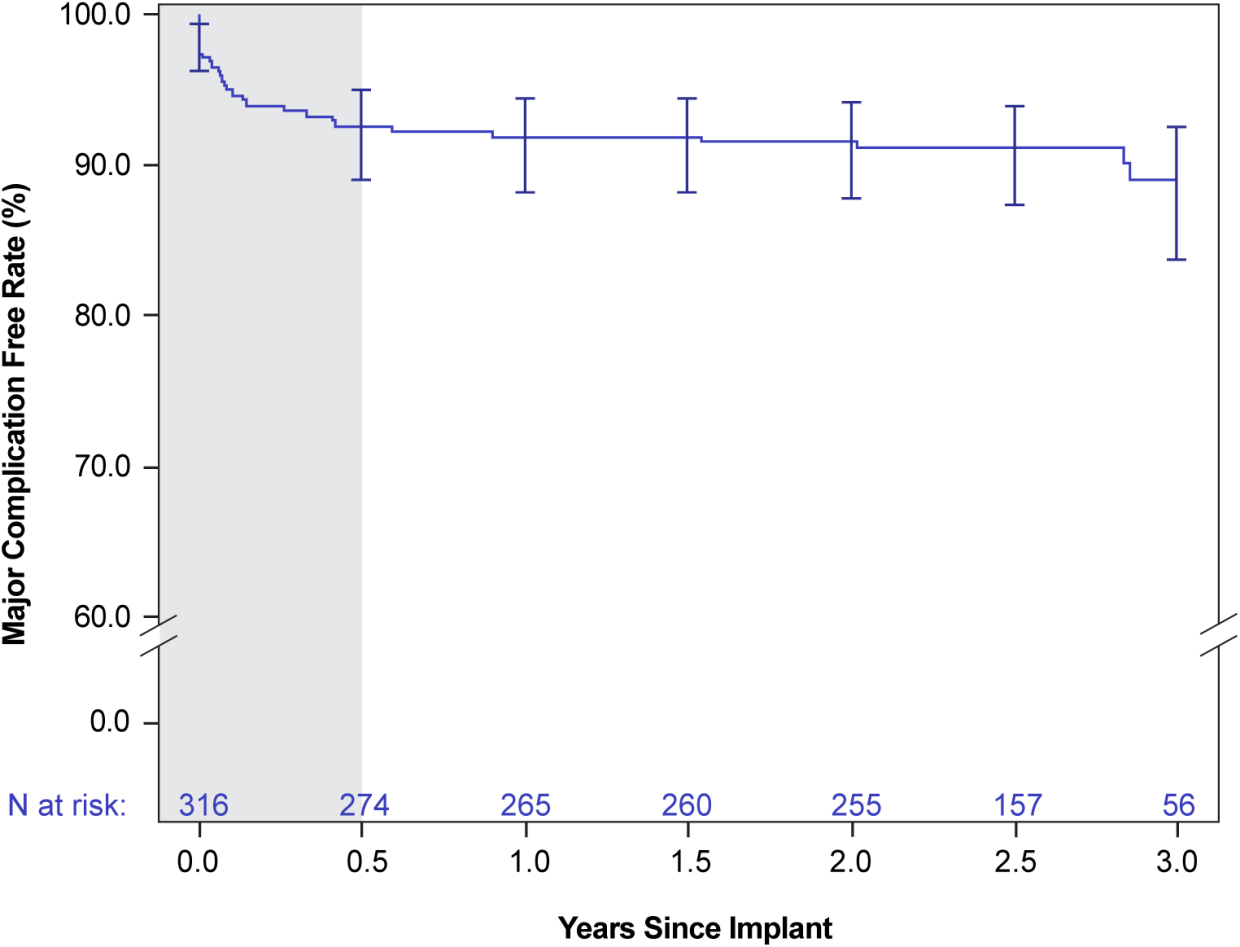
**Freedom from system- or procedure-related complications through 3 years.** Kaplan-Meier–estimated freedom from system- or procedure-related major complications through 3 years after implantation. Number at risk is represented in blue; 95% CIs are given at 6-month intervals. The 6-month time frame is shaded in gray.

A total of 31 major complications causally related to the EV ICD system or procedure occurred in 29 patients (9.2%), 6 (n=6 patients) of which occurred >6 months after implantation (Table S3). The most common were lead dislodgement (10 events; n=9 patients, 2.8%), postoperative wound or implantation site infection (n=8, 2.5%), and device inappropriate shock delivery resulting in hospitalization (n=3, 0.9%) or system revision (n=1, 0.3%; Table 2). Rate of freedom from major system- or procedure-related complications was 91.9% and 89.0% at one and 3 years, respectively (Figure 2). Three lead fractures occurred at 7, 11, and 34 months after implantation. All 3 were discovered through high-voltage lead impedance alerts. No inappropriate shocks occurred as a result of the fractures (Supplemental Results). No major intraprocedural complications, and no unique major complications related to the EV ICD system or procedure were reported. No deaths occurred from arrhythmia as a result of ineffective device therapy, and none occurred that had a causal relationship with the EV ICD system or procedure. In sudden cardiac death cases with a lack of information (eg, no device data, no autopsy) available for adjudication, events were conservatively adjudicated as possibly related to the system. Per this definition, 2 deaths were adjudicated as possibly related to the system (Supplemental Results and Table S4).

**Table 2.**
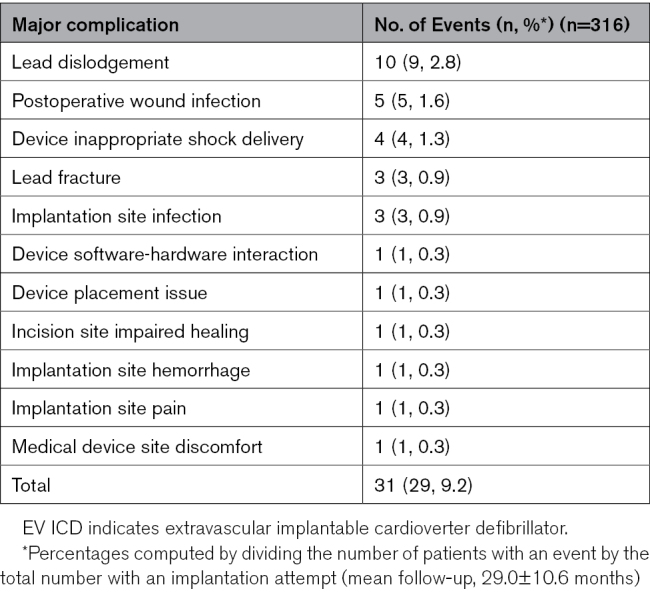
Summary of EV ICD System- or Procedure-Related Major Complications

### System Revisions

A system revision was required in 24 patients (7.6%) because of a major complication related to the EV ICD system or procedure, 19 of which occurred within 1 year of implantation (Supplemental Results). Reasons for the 24 system revisions included the following: lead dislodgement (n=9), wound or implantation site infection (n=7), lead fracture (n=3), hemorrhage (n=1), inappropriate shock (n=1), implantation site discomfort (n=1), device placement issue (n=1), and device software-hardware interaction (n=1). Of the 24 revisions, 6 involved a repositioning of the lead (n=3) or generator (n=3), whereas 18 were a removal of the lead only (n=7), generator only (n=1), or the entire system (n=10). The lead or generator was replaced with a new EV ICD lead or generator in all 8 cases in which only one or the other was removed. In patients in whom the entire system was removed, 2 were reimplanted with a new EV ICD system, and one was reimplanted with a transvenous system; in 7 cases, device reimplantation status was not available because the patient exited the study before possible reimplantation. More information on all revisions and lead removal after implantation can be found in the Supplemental Material.

### Infection

A system- or procedure-related infection was reported in 15 patients through the last follow-up, 13 occurring within 2 months of implantation (median occurrence, 0.9 months after implantation; minimum, 0.3 month; maximum, 24.2 months). Eight infections were classified as a major complication, 3 as a minor complication, and 4 as observations. The infections were related to the lateral pocket (n=9), subxiphoid incision (n=4), or both (n=2). Antibiotics, with or without wound care, were used to treat all 15 infections; 6 also required system removal, and one also required device repositioning. All 15 patients recovered without any lasting effects. There were no reports of system- or procedure-related mediastinitis, sepsis, or endocarditis, and no deaths from infection were reported.

### Inappropriate Shock

**Figure 3.**
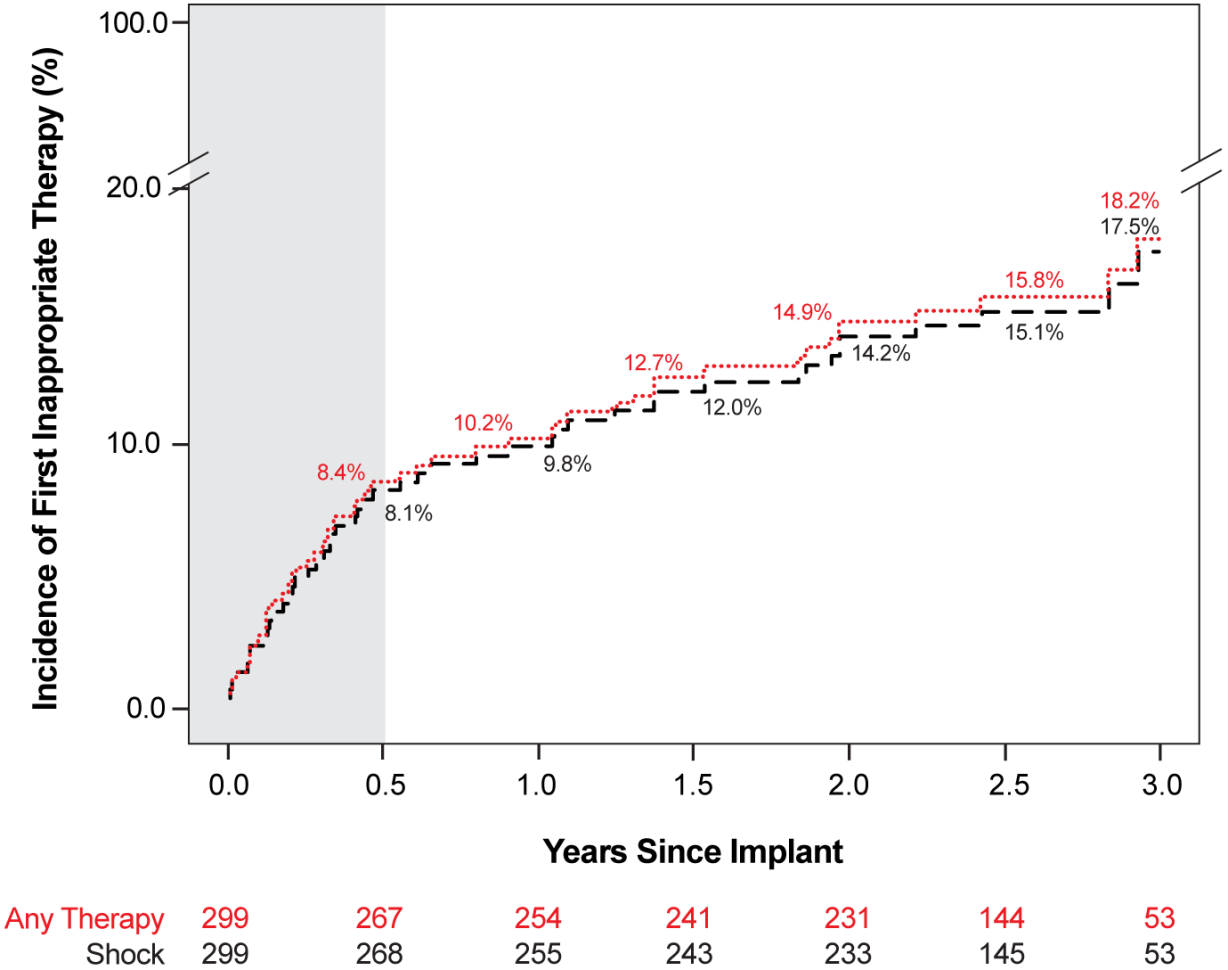
**Cumulative rate of first inappropriate therapy through 3 years.** Kaplan-Meier–estimated time to first inappropriate shock or any therapy through 3 years. Number at risk is represented below for any therapy (red) and shock (black). The 6-month time frame is shaded in gray.

Inappropriate shocks were received by 46 patients for 135 episodes through all of follow-up. The Kaplan-Meier–estimated first inappropriate shock rate was 9.8% at 1 year and 17.5% at 3 years, with the majority of first inappropriate shocks observed within 6 months of implantation (Figure 3). The most common causes of inappropriate shock were P-wave oversensing (69 episodes), myopotential noise (35 episodes), and atrial fibrillation or atrial flutter (14 episodes; Table S5). Inappropriate shocks were managed without system revision in 41 of 46 patients, of whom 30 of 41 (73.2%) did not receive a subsequent inappropriate shock after device interrogation through the final follow-up (mean, 19.0±11.7 months of follow-up after interrogation). Five patients had a system revision after inappropriate shock (lead replaced), 4 in whom lead dislodgement was the primary cause and one with chronic myopotential oversensing.

### Electrical Performance

**Figure 4.**
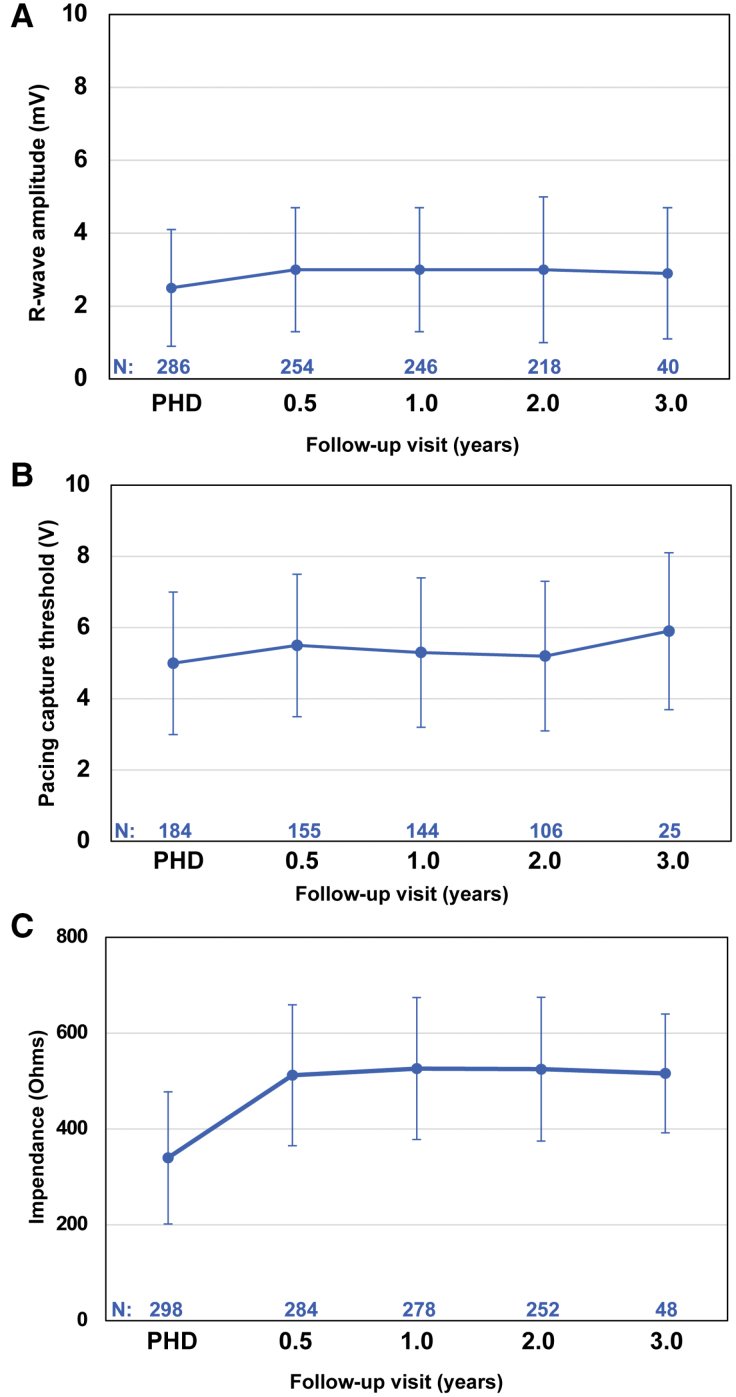
**EV ICD electrical performance through 3 years.** The mean R-wave amplitude in millivolts (**A**), mean pacing capture threshold in volts (**B**), and mean impedance in ohms (**C**) are depicted at prehospital discharge (PHD) and 0.5, 1, 2, and 3 years of follow-up with SD. The amplitude was recorded using ring 1 to ring 2 vector in an upright, sitting position; pacing threshold was recorded using ring 1 to coil 2 vector. In **B**, measurements were taken for those with successful capture at time of measurement; the mean ring 1 to coil 2 pulse widths at PHD and 0.5, 1, 2, and 3 years were 3.0±2.0, 3.4±2.3, 3.3±2.1, 3.2±2.1, and 4.6±2.8 milliseconds, respectively. Number of patients measured at each time point is given below in blue. EV ICD indicates extravascular implantable cardioverter defibrillator.

The R-wave amplitude, measured at the ring 1–ring 2 vector in a sitting position, increased after discharge but was steady over time with mean measures of 2.5±1.6, 3.0±1.7, and 2.9±1.8 mV at PHD, 0.5 year, and 3 years, respectively (Figure 4A). For those that completed capture testing, mean pacing capture threshold values were 5.0±2.0, 5.5±2.0, and 5.9±2.2 V at PHD, 0.5 year, and 3 years, respectively, for the ring 1–coil 2 vector (Figure 4B). Mean pacing impedance from ring 1–ring 2 vector increased from PHD (339.6±137.7 Ω) to 0.5 year of follow-up (512.1±146.8 Ω) but leveled off (515.8±124.1 Ω at 3 years; Figure 4C). These measures remained consistent across different vectors.

## DISCUSSION

In the initial report of this prospective, single-arm, multicenter, global study, the EV ICD system displayed defibrillation efficacy and a favorable safety profile at 6 months.^11^ We now report continued safety and effective termination of spontaneous ventricular episodes through 3 years. There were no major intraprocedural complications, underscoring the effectiveness of device-specific implantation training and purpose-built implantation tools. The majority of major system- or procedure-related complications occurred within the first 6 months. Furthermore, no major complications occurred with the EV ICD that have not been seen in other types of ICD systems. With a lead placed in close proximity to the myocardium, ATP and pause prevention pacing were successfully delivered without the need for an intravascular lead. ATP was successful in 77% of episodes in which it was used, and 2 patients received pause prevention pacing for appropriately detected episodes of prolonged asystole (Supplemental Results). The sustained effectiveness and stable electrical parameters during prolonged follow-up suggest that therapy delivery is successful across time-dependent factors such as body position, time of day, or physiological changes in the patient.

### ATP Performance

As with transvenous ICDs, the EV ICD can provide ATP, postshock pacing, and defibrillation therapies with one device. The EV ICD delivered a 77% ATP success rate, in line with the 52% to 80% success rate reported for transvenous systems.^16,17^ ATP is not available with the S-ICD, but a 2-device S-ICD and leadless pacemaker communicating system is under investigation and recently reported a 61.3% ATP success rate at 6 months.^16–18^ However, the cumulative risk associated with implantation and long-term management of 2 devices is currently unknown.

ATP therapy with the EV ICD system prevented shocks in nearly half of spontaneous VT/ventricular fibrillation episodes. This result highlights a major benefit of ATP, which is to avoid potentially unnecessary shocks that are painful, reduce patient quality of life, and increase morbidity.^19^ Although ATP has been shown to effectively treat VT, ATP is not as effective at terminating ventricular fibrillation.^17,20^ Unsuccessful ATP can result in delaying shock therapy or acceleration of ventricular arrhythmias into faster VT or ventricular fibrillation. An ATP-induced acceleration rate of 2% to 5% has been reported for transvenous systems, but there is no evidence to suggest that the rate is different with EV ICD.^17,21^ However, further evaluation of these events with the EV ICD system is needed.

A small percentage of Pivotal patients (2.8%) had ATP programmed “off” at 2 years because of sensation related to in-office electrical testing, and no patients who received successful ATP for spontaneous episodes had ATP subsequently programmed “off.” In addition, the proportion of patients with ATP programmed “on” increased over the course of the study. Taken together, these results illustrate that in-office testing may not reflect the ambulatory experience with ATP and that, as a whole, ATP therapy from an EV ICD is well tolerated and accepted by patients as a measure to terminate ventricular episodes and potentially reduce shock burden.

### Safety

The EV ICD system exhibited a 92.6% rate of freedom from major system- or procedure-related complications at 6 months and 89.0% rate at 3 years. This rate is on par with both the 88.7% 3-year rate reported for the S-ICD in the EFFORTLESS study (Evaluation of Factors Impacting Clinical Outcome and Cost Effectiveness of the S-ICD) and rates reported for transvenous systems (90.9% through 16 months), although a direct comparison would be needed to definitively determine how the different ICDs compare with one another in terms of safety.^22,23^ There were 24 system- or procedure-related major complications that resulted in a system revision, 19 of which occurred within 1 year of implantation. Despite the EV ICD lead being placed close to the heart, there were no cardiac injuries during implantation or through follow-up. Lead dislodgements (10 events; n=9 patients, 2.8%) were the most frequent major complication in the Pivotal study; all occurred within 6 months of implantation and were attributed to suboptimal suturing or improper lead placement. As a result, implantation training emphasizes a minimum of 3 sutures to fixate the lead and tunneling left of the sternal midline for proper lead placement. For context, acute lead dislodgement is reported to be 2% to 8% over the long term with transvenous systems (1.3% predischarge rate) but is less frequent with S-ICDs (<1%).^8,24,25^ Lead fractures were noted in 3 patients (attributed to suboptimal lead placement in 2 patients) and have been addressed with training improvements and manufacturing enhancements. Future evaluation of real-world EV ICD performance will be needed to assess the effect of these refinements through the life of the device. Pivotal study participants had the opportunity to continue follow-up by enrolling in the postapproval Enlighten study, so extended clinical data will be available for a significant proportion of Pivotal patients for the life of the device.

Fifteen infections occurred that were related to the EV ICD system or procedure (2 occurring >6 months after implantation), 8 of which were classified as a major complication. A majority were successfully treated conservatively with antibiotics and wound care only, and 7 (2.2%) also required a system revision. All infections resolved after treatment, and no potentially life-threatening infections, including mediastinitis, sepsis, or endocarditis, related to the system or procedure occurred. When lead removal was necessary, it was accomplished with simple traction in most cases or with commonly available lead extraction tools (Supplemental Results). Most lead removals in this study occurred within 1 year of implantation; continued observation of chronic EV ICD lead extraction is needed.

### Inappropriate Shocks

The 1-year and 3-year inappropriate shock rates for the EV ICD were 9.8% and 17.5%, respectively. In comparison, the 1-year inappropriate shock rate for the S-ICD was 13.1% in its investigational device exemption trial.^7^ More recently, S-ICD has reported improved 1-year inappropriate shock rates of 3.1% in the UNTOUCHED study (Understanding Outcomes With the S-ICD in Primary Prevention Patients With Low Ejection Fraction), although secondary prevention patients were excluded, and 6.7% in a postapproval study.^26,27^ P-wave oversensing was the most common reason for inappropriate shock with the EV ICD (51% of episodes) attributable to lead placement near the right atrial appendage, which is not a main culprit of inappropriate shock with other ICDs. The current commercially available Aurora EV-ICD system now includes a novel algorithm (Smart Sense) aimed at reducing P-wave oversensing, which was not available at any point during the Pivotal study. In a retrospective simulation of EV ICD Pivotal study episodes, Swerdlow et al^28^ showed that the Smart Sense algorithm reduced the total inappropriate shock rate by 29% with no impact on sensitivity. According to these findings, the results presented herein, particularly in regard to inappropriate shock, may not be representative of the real-world experience with the Aurora EV-ICD system.

Incorporation of novel algorithms is one method aimed at lowering inappropriate shock rates. They can also be mitigated through patient-tailored programming and refinement of surgical technique and lead positioning with added implanter experience. Because of these improvements, inappropriate shocks tend to decrease with time, as has been observed with transvenous and subcutaneous devices.^29,30^ In the present study, >70% of patients who received a first inappropriate shock did not experience a subsequent inappropriate shock after device interrogation, suggesting an opportunity for programming optimization. The first inappropriate shock rate slowed after 6 months, with a majority of the first inappropriate shocks occurring during the first year after implantation. Device programming changes used to address inappropriate shock included changing the sensing vector, extending the time to detection, and increasing the sensing amplitude; other strategies used to manage inappropriate shock included medication adjustments or device revision.

### Substernal Lead Performance

As previously reported, substernal lead placement resulted in effective defibrillation that remained stable over time in patients who completed chronic defibrillation threshold testing.^11^ At implantation, defibrillation testing was successful at ≤30 J in 98.7% of patients and was successful in all patients who underwent testing at 6 months. In addition, shocks were successful for 100% (27/27) of discrete, spontaneous episodes in 17 patients; however, assessment of shock success for ambulatory discrete episodes will be needed in a larger cohort of patients. The shock energies required for successful defibrillation with EV ICD are in accordance with defibrillation thresholds used in transvenous systems and lower than what has been required for the S-ICD.^7^ Pacing and electrical performance of the EV ICD were steady over time, demonstrating the stability of leads placed substernally. R-wave amplitudes increased slightly after PHD but then stabilized through the 3-year follow-up and were similarly stable regardless of body positioning or vector configuration. Pacing capture thresholds and impedance were stable after discharge, but pacing thresholds were higher than what is expected with a transvenous system implanted within the heart. Among those who had postshock or pause prevention pacing programmed “on” and required such therapy, 3 received successful pacing (Supplemental Results).

### Patient Experience

In addition to effective therapy, the EV ICD system has demonstrated positive patient experience in the Pivotal study. The lower energy needed for defibrillation with EV ICD allows a projected battery longevity that is comparable to that of transvenous systems; Knight and colleagues^12^ previously showed, using modeling, that the extended battery life could reduce the number of device replacements and long-term cost for the patient. However, pacing thresholds for EV ICD are higher than what is typical of transvenous systems, which could negatively affect battery longevity in patients who frequently receive pacing therapy. The EV ICD Pivotal study previously reported improvements in patient physical quality of life from baseline to 6 months.^31^ Specifically, Sears et al^31^ found favorable quality of life results for patients with the EV ICD system compared with previous studies of other systems using the same metrics (Florida Patient Acceptance Survey Score). Real-world experience will be needed to further characterize the holistic patient experience with EV ICD to determine whether improved patient quality of life, in addition to clinical outcomes, persists through the lifetime of the device.

### Limitations

This study must be considered within the context of its limitations. The study was nonrandomized and single arm, with no comparator to subcutaneous or transvenous systems. Procedures were performed at expert centers in a clinical trial environment with a prespecified follow-up and testing protocol. Because the population in this study was younger than those who might typically receive an ICD, more data will be needed to evaluate the EV ICD performance in older patients with more comorbidities. These results display multiyear efficacy and safety, but assessing the EV ICD system over the lifetime of the device will be critical. This includes a more robust assessment of lead removal, device longevity, long-term complications, therapy rates, and lead stability over long (>4 years) time frames. As previously discussed, the EV ICD system implanted during the Pivotal study differs from the one currently being implanted because of the addition of an inappropriate shock reducing algorithm (Smart Sense) and manufacturing enhancements. In addition, implanter training has since been updated to focus on avoiding dislodgement and minimize P waves, so these results, inappropriate shocks in particular, may not be applicable to current experience. The Enlighten Study, a global, prospective registry, will evaluate the real-world safety and performance of the Aurora EV-ICD system with the Smart Sense algorithm over the lifetime of the device (ClinicalTrials.gov identifier: NCT06048731).

### Conclusions

In this prospective global study, the EV ICD system terminated spontaneous ventricular arrhythmias with a high rate of ATP and defibrillation therapy success and a low major complication rate through long-term follow-up. The EV ICD system, with substernal lead placement, can provide ATP and low-energy defibrillation in a single device while outside the vasculature.

## ARTICLE INFORMATION

### Acknowledgments

The authors thank Thomas Holmes, PhD, of Medtronic Inc for help with preparing the manuscript.

### Sources of Funding

This study was fully supported by Medtronic, Inc.

### Disclosures

Dr Friedman reports receiving EV ICD Pivotal study participation support, paid to their institution, from Medtronic; licenses and patents with Anumana, Eko Health, Alive Cor, and Marani Health; advisory board participation at Anumana; a leadership position with xAI.health and MediCool; steering committee participation and advisory group participation at Boston Scientific, funds paid to their institution; and stock from MediCool, Marani Health, and xAI.health. Dr Murgatroyd reports receiving EV ICD Pivotal study participation support, paid to their institution, from Medtronic; consulting fees from Medtronic for advisory board and steering committee participation, consulting fees from Boston Scientific for steering committee participation; and speaker fees from Medtronic. Dr Boersma reports receiving EV ICD Pivotal study participation support, paid to their institution, from Medtronic and consulting fees from Medtronic and Adagio, paid to the cardiology department at their institution. Dr Manlucu reports receiving EV ICD Pivotal study participation support, paid to their institution, from Medtronic; consulting fees from Medtronic for advisory board and steering committee participation; and speaker fees from Medtronic. Dr Knight reports receiving EV ICD Pivotal study participation support, paid to their institution, from Medtronic; consulting fees from Medtronic for advisory board and steering committee participation; consulting fees from Boston Scientific for advisory board participation; and speaker fees from Medtronic and Boston Scientific. Dr Clémenty reports receiving consulting fees, honoraria fees, travel support, and advisory board participation from Medtronic. Dr Leclercq reports receiving consulting fees and honoraria from Medtronic, Boston Scientific, and Abbott for steering committee participation and speaker fees from Medtronic. Dr Amin reports receiving EV ICD Pivotal study participation support from Medtronic; an institutional grant from Biosense Webster; consulting fees from Medtronic, Boston Scientific, Biosense Webster, Atricure, and Philips; honoraria from Medtronic, Boston Scientific, Biosense Webster, Atricure, and Philips; travel support from Boston Scientific, Medtronic, and Philips; and advisory board participation at Boston Scientific, Medtronic, Atricure, and Philips. Dr Merkely reports receiving institutional grants from Medtronic and Boston Scientific and lecture fees from Medtronic, Biotronik, and Abbott. Dr Birgersdotter-Green reports receiving honoraria from Medtronic, Boston Scientific, Abbott, and Biotronik; advisory board participation at Biotronik; and stock ownership in Vektor Medical. Dr Chan reports receiving honoraria for lectures from Medtronic. Dr Biffi receiving EV ICD Pivotal study participation support, paid to their institution, from Medtronic and grants from Medtronic, paid to their institution. Dr Knops reports receiving EV ICD Pivotal study participation support from Medtronic for trial funding; grants from Boston Scientific and Abbott for trial funding; institutional and private consulting fees from Abbott and Boston Scientific; honoraria from Medtronic, Boston Scientific, and Abbott; advisory board participation at Boston Scientific, Atacor, and Kestra; participation on the ESC pacing guideline committee; and stock ownership from Atacor. Dr Engel reports consulting fees from Medtronic. Dr Epstein reports institutional support for the EV ICD Pivotal study, consulting fees from Medtronic, and honoraria from Medtronic. Dr Johansen reports honoraria paid to their institution from Merit Medical, Education course and advisory board participation at Medtronic and Biotronik. Dr Sterliński reports receiving EV ICD Pivotal study participation support, paid to their institution, from Medtronic; grants from Biotronik and HammerMed; consulting fees from Abbott, Biotronik, HammerMed, and Medtronic; honoraria from Abbott, Biotronik, Medtronic, and Zoll; and advisory board participation at Medtronic. Dr Hounshell reports consulting fees from Medtronic, lecture fees from Medtronic, and advisory board participation at Medtronic. Dr Abben reports consulting fees and honoraria from Medtronic. A. Thompson, Y. Zhang, Dr Wiggenhorn, and S. Willey report employment by Medtronic. Dr Crozier reports receiving research funding for the EV ICD Pivotal study from Medtronic, grants from Medtronic for fellowship funding, and consulting fees from Medtronic. The other authors report no conflicts.

### Supplemental Material

Supplemental Methods

Supplemental Results

Tables S1–S5

Figures S1 and S2

Reference 32
